# Evidence for the Re-Enactment of a Recently Learned Behavior during Sleepwalking

**DOI:** 10.1371/journal.pone.0018056

**Published:** 2011-03-21

**Authors:** Delphine Oudiette, Irina Constantinescu, Laurène Leclair-Visonneau, Marie Vidailhet, Sophie Schwartz, Isabelle Arnulf

**Affiliations:** 1 Sleep Disorders Unit, Pitié-Salpêtrière Hospital, APHP, Paris, France; 2 Inserm, U975, Paris, France; 3 Université Pierre et Marie Curie-Paris6, Centre de Recherche de l'Institut du Cerveau et de la Moelle epiniere, UMR-S975, Paris, France; 4 Department of Neuroscience, and Geneva Neuroscience Center, University of Geneva, Geneva, Switzerland; 5 Swiss Center for Affective Sciences (NCCR), University of Geneva, Geneva, Switzerland; University of Oxford, United Kingdom

## Abstract

Animal studies have shown that sequenced patterns of neuronal activity may be replayed during sleep. However, the existence of such replay in humans has not yet been directly demonstrated. Here we studied patients who exhibit overt behaviors during sleep to test whether sequences of movements trained during the day may be spontaneously reenacted by the patients during sleep.

We recruited 19 sleepwalkers (who displayed complex and purposeful behaviors emerging from non REM sleep), 20 patients with REM sleep behavior disorder (who enacted their dreams in REM sleep) and 18 healthy controls. Continuous video sleep recordings were performed during sleep following intensive training on a sequence of large movements (learned during a variant of the serial reaction time task).

Both patient groups showed learning of the intensively trained motor sequence after sleep. We report the re-enactment of a fragment of the recently trained motor behavior during one sleepwalking episode.

This study provides, to our knowledge, the first evidence of a temporally-structured replay of a learned behavior during sleep in humans. Our observation also suggests that the study of such sleep disorders may provide unique and critical information about cognitive functions operating during sleep.

## Introduction

It is well established that sleep facilitates plastic changes that underlie the consolidation of recently acquired knowledge [1], [2], [3], [4]. The prevailing hypothesis states that the neural traces coding for the newly acquired information are reactivated during sleep, thus fostering memory consolidation. In rats and birds, specific patterns of neural activity associated with recent waking behavior are spontaneously replayed during subsequent sleep [5],[6]. Similarly, functional neuroimaging studies in humans have shown that brain regions involved in motor skill learning are reactivated during post-training sleep [7]. These regional increases in cerebral blood flow could correspond to the replay during sleep of patterns of neural activity coding for newly acquired information, as observed in animals, or reflect other experience-dependent brain processes, such as local homeostasis [4]. Replay during sleep is also suggested by two recent studies in which sensory cues, previously associated with learned material, were presented during sleep, yielding better memory performance [8], [9]. Dreams also contain a high proportion of recent waking experiences [10], [11], [12]. However, direct evidence for a replay of temporally-structured information during human sleep is still lacking.

Here we propose to address this question by studying patients who exhibit overt behaviors while asleep, such as patients with REM sleep behavior disorder or sleepwalkers. REM sleep behavior disorder is a recently-discovered parasomnia characterized by a loss of the physiological REM sleep-associated muscle atonia [13], which results in motor activity reflecting the enactment of violent and vivid dreams [13], [14]. The behaviors during REM sleep behavior disorder are various, purposeful and complex [14], [15], [16], [17]. Chronic REM sleep behavior disorder mainly affects middle-aged men, as an isolated condition (idiopathic REM sleep behavior disorder) or associated with narcolepsy and neurodegenerative diseases (mainly synucleinopathies) [18]. Sleepwalking affects mostly children and young adults and is characterized by overt and complex motor behaviors initiated during slow-wave sleep [13]. Dreaming during sleepwalking is sometimes reported in adults, which constitutes a form of dream-enacting behavior much like REM sleep behavior disorder [19].

In the present study, we trained both patients with REM sleep behavior disorder and sleepwalkers on a modified version of a serial reaction time task, which is known to robustly benefit from sleep [7], [20], [21]. We tested whether, during sleep, the patients would replay fragments of a recently trained sequence involving large arm movements.

## Methods

### Ethic Statement

The institutional review board 06-03 of the ethics comittee (Comité de Protection des Personnes Ile de France 06) gave its approval for the study, which was considered as non invasive.

For patients with REM sleep behavior disorder and sleepwalkers, the cognitive test was proposed to the subjects before and after a normal videopolysomnograpy procedure, scheduled for diagnosing their disease. Hence it was considered as a minor, non invasive deviation of routine clinical care in these diseases by the ethics committee, requiring an oral consent of the patient (the collection of the oral consent was consigned in the medical file by the physician in charge). As for healthy subjects, a written informed consent was obtained.

The patient and the healthy control who appear in the Video S1 and in the Figures have seen this manuscript and have provided written consent for publication in a scientific journal.

### Subjects

A total of 20 patients with REM sleep behavior disorder (mean age 66.5±6.5 years; 16 males, 4 females), 19 sleepwalking patients (mean age 34.4±15.4 years; 6 males, 13 females), and 18 healthy controls (mean age 57.9±5.3 years; 14 males, 4 females) took part in this study. As expected from these pathologies, the population of sleepwalkers was younger than the population of REM sleep behavior disorder patients (p<0.001). We also tested a group of healthy controls whose mean age would lie between those of the patient groups. It is important to note that the inclusion of the control group mainly serves to confirm that the sequence of ample movements could be learned equally by all these distinct populations. The critical measures of learning involve within-subject comparisons, while differences in mean reaction-times due to age are expected but not relevant here.

REM sleep behavior disorder was defined according to standard criteria [13], including i) a positive clinical history (bed partners reporting most often violent, purposeful limb or body movements, as if the patients were acting out their dreams), and ii) the presence of abnormally enhanced chin or phasic muscle tone and/or complex and non-stereotyped movements (such as gesturing, reaching, grabbing, punching, kicking, talking, laughing, running, chewing, feeding, drinking) during REM sleep on video sleep recording. In the sleep behavior disorder group, 13 patients had idiopathic REM sleep behavior disorder and 7 patients had Parkinson disease, with a mild to moderate motor disability (Hoehn and Yahr score between I and III) [22], which did not impact their daily activities, and no dementia (Mini-Mental State Examination score higher than 23) [23].

Sleepwalking was defined according to clinical international criteria [13], plus at least one arousal during slow-wave sleep associated with a motor episode suggestive of surprise, confusion, or fear (startle response, sitting up in the bed, looking around surprised), or numerous sudden arousals during slow-wave sleep and no epilepsy or sleep-disordered breathing. We included in the study primary sleepwalking patients, without any pre-existent psychiatric and/or neurological illness.

Subjects were informed that we tested their performance in the task execution, but they were not aware of the regularity of the trained sequence, nor that we expected a potential replay during sleep.

All participants gave informed consent to participate in this study, which was approved by the local ethics committee. Healthy volunteers (but not the patients) were paid for their participation.

### Behavioral task and experimental procedure

The subjects were trained on a modified version of the serial reaction time task [24], which was previously shown to elicit sleep-related performance improvement [7], [20], [21]. Instead of requiring finger movements, the present task involved large hand, forearm and arm movements that would be clearly visible on the video recorded during sleep. Note that twitches of finger muscles are extremely frequent during normal REM sleep, and even more during REM sleep behavior disorder [25], so that a replay of learned fingers movements could not be easily distinguished from multiple and non-specific twitches during the REM sleep.

**Figure 1:**
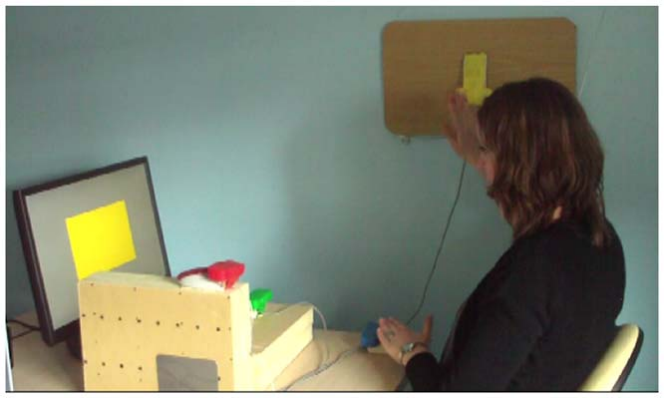
Experimental design. The subjects were trained on a modified version of a serial reaction time task. Four colored response buttons were positioned at distinct spatial locations and the subjects had to press on the response button matching the color shown on a computer screen. The subjects were intensively trained on a fixed eight-item sequence (Blue-Yellow-Green-Red-Yellow-Blue-Red-Green). They were asked to use their left hand for the blue and green buttons and the right hand for the red and yellow buttons. The task involved a sequence of ample and uncommon movements (e.g., crossing arms, pronated forearm, flexed and extended hand) that would be unambiguously recognized if replayed during sleep.

The subjects sat comfortably facing the computer screen; they were asked to react as quickly and accurately as possible when a large, colored rectangle appeared on the screen by pressing the corresponding colored response button (e.g., when the rectangle on the screen was green, the subject had to press the green button). The four response buttons were attributed four different colors (red, yellow, blue, or green) and were placed at different spatial localizations, as shown in Figure 1. The subjects were asked to use their left hand for the blue and green buttons and their right hand for the red and yellow buttons so that all participants carried out the same sequence of movements, including movements crossing the midline (see Video S1).

Each correct response was immediately followed by the presentation of the next stimulus on the screen, thus eliciting a new response, and so on. If the subject failed to press the appropriate button, visual (a white rectangle) and auditory (a simple beep) feedback were simultaneously delivered followed by the presentation of the next visual stimulus. We used E-Prime (Psychology Software Tools, Inc.) for stimulus presentation and response recordings.

The subjects were intensively trained on a fixed 8-item sequence (Blue-Yellow-Green-Red-Yellow-Blue-Red-Green) and were not informed about the regularity of the sequence. To distinguish between structured sequence learning and improvement in visuomotor responses independent of the sequence, a random sequence was also presented. Like the structured sequence, the random sequence was composed of two series of four colors, in which each of the four buttons was pressed once, but in a completely random order. Because each color appeared twice in each eight-item sequence of the random condition, only the order of the colors was altered between the random and the structured conditions. Subject performance was quantified by measuring reaction time and response accuracy (correct hand and correct button use).

We developed this version of the serial reaction time task optimize the detection of spontaneous replay during a subsequent REM sleep behavior or a sleepwalking episode. In particular, the task involved ample movements, with the response buttons placed about 50 cm apart in the peripersonal space, and the movements executed during the task were unusual as compared to the behaviors generally observed during parasomnia episodes (our task included pronating forearm and flexing hand movements, as well as arms crossing the midline). Video S1 shows the sequence performed by a healthy control subject during a training session and, after acquisition, while lying in a bed and performing the sequence from memory to facilitate later visual comparison with any potential replay during sleep.

The experimental procedure consisted of a training session at 6 pm and an initial test session at 8 pm, followed by a night of sleep. The training session consisted of four consecutive blocks of structured sequences (10 sequences in each block, 80 trials per block). The initial test session consisted of four such blocks and one additional block of random sequences placed in the middle of the session (third block of the five-block session). The following morning, the subjects were retested at 9 am with the same series of blocks as during the initial test session. In this protocol, each subject performed a total of 1,120 trials.

**Figure 2:**
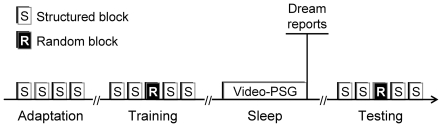
Experimental procedure. The experimental procedure included one training session immediately followed by an initial testing session in the evening, and one testing session the following morning after one night of sleep. The training session consisted of four consecutive blocks of ten repetitions of the structured sequence. The testing sessions before and after sleep consisted each of four blocks of ten repetitions of the structured sequence, with one block of ten random sequences.

On the morning immediately after awakening from sleep and before the test session, the participants were asked about their night dreams the previous night. A summary of the experimental procedure is provided in Figure 2.

### Sleep and nocturnal behavior monitoring

All subjects underwent video and sleep monitoring during the night immediately following the training; 7 sleepwalkers and 7 patients with REM sleep behavior disorder were recorded over two consecutive nights, with no additional training or retesting in between the two nights. Scalp electroencephalography included three bipolar channels for the patients with REM sleep behavior disorder and the controls (Fp1/Cz, O2/Cz, C3/A2) and eight bipolar channels for sleepwalkers (FP1/C3, C3/O1, C3/T3, T3/O1, FP2/C4, C4/O2, C4/T4, T4/O2) to exclude nocturnal frontal lobe epilepsy. Monitoring also included EEG-synchronized infrared video-monitoring and sound recording in the room (Brainet®, Medatec Ltd, France), a right and left electro-oculogram, submentalis and tibialis anterior muscle electromyogram, nasal pressure through a cannula, tracheal sound recording through a microphone placed at the surface of the trachea, thoracic and abdominal strain jauges to assess respiratory efforts, electrocardiography, and pulse oximetry. Sleep stages, EEG arousals, muscle activity, periodic leg movements and respiratory events were scored by visual inspection of the multimodal recordings, using standard criteria [26], by two independent and experienced scorers.

### Statistical analyses of motor performance

Statistical analyses of behavioral measurements were performed using Statistical Package for Social Sciences (SPSS Inc, Chicago, IL, USA). For each of the three groups of subjects (sleepwalking group, REM sleep behavior disorder patients group and control group) we performed a repeated-measures ANOVA with sessions (training, testing) and type of sequence (structured, random) as factors. For the type of sequence factor, we selected the random block and the structured block performed just afterwards, because they were close in time (respectively block 3 and block 4), which was important given the fatigability of some of the patients. The selected blocks were also contextually very similar (both blocks implied a switch of sequence type, from structured to random and from random to structured sequence). In these analyses, we considered each group separately because the subjects in the different groups were not matched for age (e.g., REM sleep behavior disorder patients were significantly older and had significantly slower reaction times than the sleepwalkers, as expected in these disorders) and because the goal of these analyses was to test for sequence-specific learning by comparing change in performance for the structured sequence and for the random sequence between the pre-sleep and post-sleep testing sessions.

### Assessment of sequence replay during sleep

To confirm the putative replay of a fragment of the sequence observed in one sleepwalker (see Results), we asked 11 independent judges blind to the aim of the experiment to assess resemblance of 113 video clips of sleepwalking episodes with a video showing an awake subject performing the task from memory while lying in a bed, in the same recording conditions as the patients (Video S1). For each video clip, the judges were asked six questions concerning the sleepwalking episode (see Text S1), including a final assessment of the resemblance of sleepwalking movements with the movements performed by the awake subject on a scale from 0 (no resemblance at all) to 10 (identical movement sequence).

Note that because we did not observe any sign of replay in the REM sleep behavior disorder, a similar procedure was not used with REM sleep behavior disorder episodes.

## Results

### Sleep and cognitive performances

Sleep recordings confirmed a normal sleep pattern in the night following training in all the three tested groups. Expected differences were found for some sleep parameters when comparing REM sleep behavior disorder patients with healthy controls (such as total sleep time and sleep efficiency, see Table 1). Of note, the duration of slow-wave sleep was longer in controls than in REM sleep behavior disorder patients (p<0.001) but remained within normal values.

**Table 1:** Sleep measures in sleepwalkers, patients with REM sleep behavior disorders (REM SLEEP BEHAVIOR DISORDER), and healthy middle-aged controls.

	Sleepwalkers	Patients with REM SLEEP BEHAVIOR DISORDER	Healthy controls
Number of subjects	19	20	18
Age (y)	34.4±15.4	66.5±6.5*	57.9±5.3
Night-time sleep			
Total sleep time (min)	488±66	376±75*	428±73
Sleep efficiency (%)	88.5±10.8	78.6±11.4*	86.4±6.6
Latency to sleep onset (min)	25.5±15.5	40.5±33.8*	22.3±16.0
Number of REM sleep episodes	4.6±0.9	3.1±1.6*	4.5±1.1
Sleep duration (% total sleep time)			
Stage N1	4.7±2.5	8.5±6.9	6.0±6.9
Stage N2	49.6±8.0	55.5±9.6*	48.3±8.6
Stage N3 (former stage 3–4, slow-wave sleep)	26.1±6.5	19.1±6.4*	25.9±6.8
REM sleep	19.5±4.1	16.8±7.9	19.6±5.2
Sleep fragmentation (No events/h)			
Arousals	13.8±7.0	19.2±9.2	20.1±9.5
Apnea/hypopnea	1.1±1.6	8.0±10.4	12.1±10.2
Periodic leg movements	4.6±6.7	33.9±37.4	14.4±29.9

*p<0.05 comparison patients with REM SLEEP BEHAVIOR DISORDER and controls.

Upon testing after one night of sleep, both groups performed better than during the pre-sleep session (Figure S1). Improvement after sleep was greater for the trained, structured sequence than for the random sequence. Furthermore, the results showed an interaction between session (pre-sleep or post-sleep) and condition (structured sequence or random sequence) (F_(1,16)_ = 9.456, p = 0.007 in controls; F_(1,18)_ = 6.670, p = 0.019 in REM sleep behavior disorder patients; F_(1,18)_ = 20.247, p<0.001 in sleepwalkers), confirming sequence-specific learning for our task, which was more complex than the classical serial reaction time task. It is already known that sleep may facilitate learning of sequences of movements [7], [20]. In the present work, our aim was not to re-validate pre-existent knowledge about sleep-related memory consolidation, but to make use of a task that may benefit from sleep to increase the probability to observe task-related behaviors during sleep.

### Dreams content after the post-training night of sleep

On the morning following each of the first night of recorded sleep, only seven subjects out of the 57 reported some dream content. Most (40/57) tested subjects did not recall any dreams, and 10 subjects reported a ‘blank dream’ (feeling of having dreamt but no memory of it). There was no difference in dream recall frequency between the controls, the patients with REM sleep behavior disorder and the sleepwalkers. Interestingly, 2 out of the 7 subjects who recalled a dream reported some content related to the recently learned task. One sleepwalker, who spontaneously used the word “cross” to verbally characterize part of the motor sequence required by the task, reported following the night of sleep, a “cross” dream involving two diagonal paths. In his dream, the patient was driving to school to register his children (this path corresponded to the first diagonal). Suddenly, an unknown driver struck the patient's parked car and fled to the airport located in the opposite direction (second diagonal). The second report of dream content related to the learned task came from a healthy control subject who dreamed that he was concerned about respecting the order of the colors in the sequential task and tapped the colored response buttons in his dream.

### Behaviors exhibited during post-training sleep

The patients with REM sleep behavior disorder exhibited several complex behaviors during REM sleep of the two experimental nights (i.e., hand movements, defense posture, kicking, punching, reaching, smiling, pointing, leaping out of bed, whispering and speaking). No obvious motor replay of the task was identified among these REM sleep-associated behaviors.

**Figure 3:**
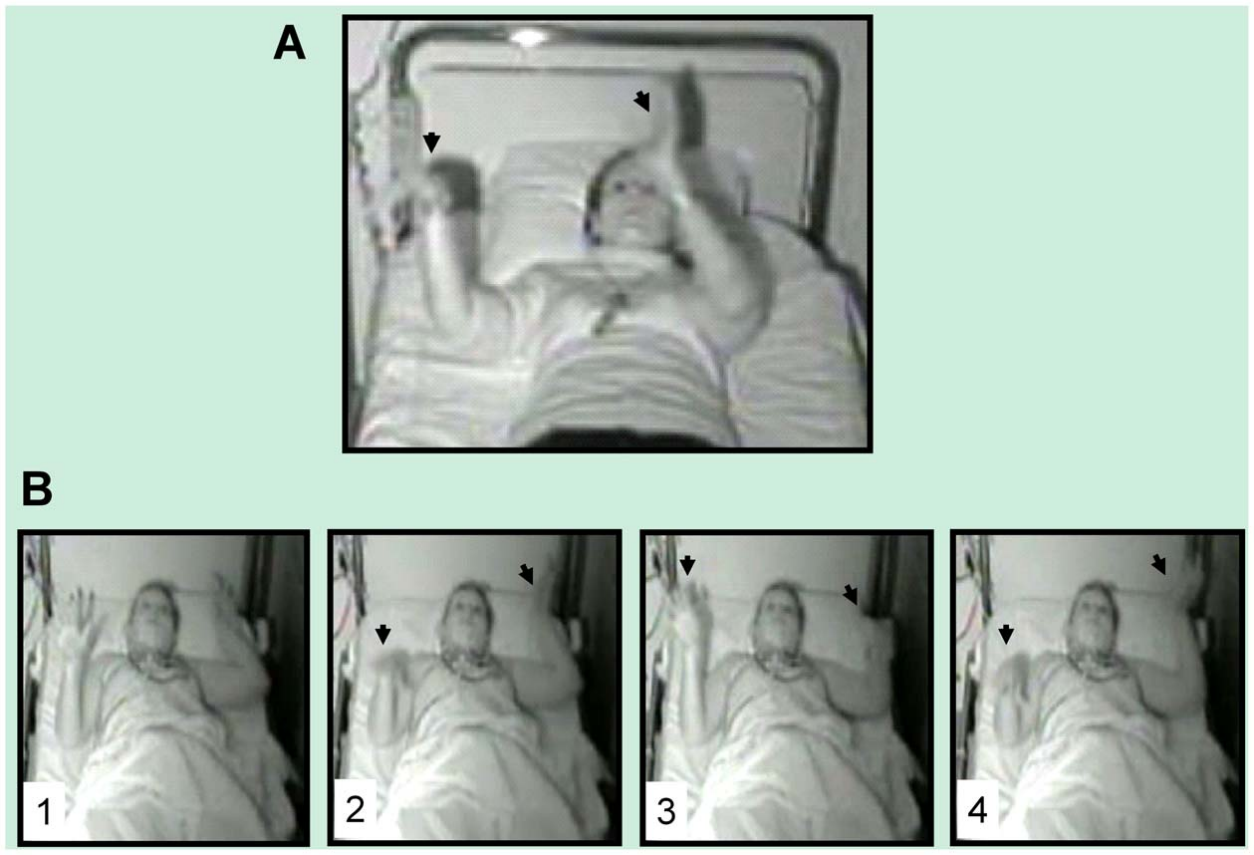
Snapshots extracted from video recordings. (A) Execution of the sequence from memory by a wake control subject lying in a bed. The subject reaches toward the imaginary location of the yellow response button (left arrow) while preparing to press the next (green) button (right arrow). (B) Overt replay of the structured sequence by a sleepwalker during slow-wave sleep. After a sudden arousal, the patient raised both arms, with pronated forearm, and waited two seconds as if preparing to perform the task (1). Then, she appeared to press imaginary response buttons sequentially with the right, left and right hand (2, 3, and 4, respectively). Note the striking similarity between the posture displayed in A and in the fourth panel in B.

**Figure 4:**
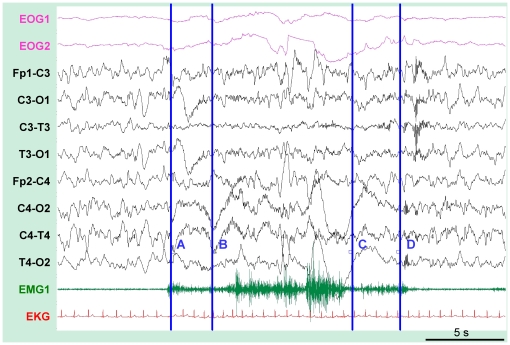
Sleep-monitoring during the overt replay of the sequence in a sleepwalker. During slow-wave sleep (non-REM stage 4), the patient first startled (A), raised her left arm (B) and then sequentially pressed invisible response buttons with the right and left hands (between C and D). During the episode, the associated EEG showed persistent slow background activity with mixed delta, theta and alpha frequencies. A mild heart rate increase was also observed on the electrocardiogram. After the replay, a spindle was observed on the EEG recording. EOG1 and EOG2, electrooculograms, FP1-C3, C3-O1, C3-T3, T3-O1, FP2-C4, C4-O2, C4-T4; T4-O2, 8 channels of electroencephalograms; EMG1, electromyogram of the submentalis muscle; EKG, electrocardiogram.

During the two recorded nights following training, the sleepwalking patients exhibited several typical behaviors of sleepwalking during slow-wave sleep (startling, looking around confused, speaking, raising the head and the chest, touching the bedside table), the part of these enacted behaviors corresponding only to a mean of one minute of non REM sleep per night. Furthermore, we observed one uncommon behavior in a sleepwalker, suggestive of a replay of the recently trained motor task: during slow-wave sleep of the second post-training night, at 00:58 am, Patient 1 initially startled, then opened her eyes, stood up, laid down again, whispered, and engaged in a sequence of hand and arm movements. She sequentially raised her left arm with her hand extended and pronated like when executing the recently learned task, raised her right arm with her fingers outspread, waited in this raised position, and slowly pressed down with the right hand, left hand and then right hand, as if pressing down invisible pads (Figure 3, Figure 4 and Video S1). The unusual behavior described in this sleepwalking patient reflected obvious and accurate re-enactment of a short fragment of the recently learned sequence of movements (as later confirmed by the independent judges, see below). The next morning, the patient had the feeling of having dreamt but could not remember any dream content.

The sleepwalker who described the cross dream (Patient 2) also displayed an uncommon behavior for a sleepwalking episode (he raised and dropped his right hand twice and his left hand once) when emerging from slow-wave sleep, before being fully awake. While the observed movements in this patient presented some analogy with the gestures of the task, they cannot be considered as a replay of the sequence itself.

### Evidence for behavioral replay during sleep

Only the putative replay of the first sleepwalker was judged resembling with the wake task movements, with an average score of 7.2±2.6 (score/10); the others movements displayed by sleepwalkers during the experimental nights were rated below 5/10 by the judges (Figure S2). Because the judges differed in the way they used the scoring scale (some being more conservative than others), the 0–10 score was converted to a 0–1 scale (1 corresponding to the maximal rate given over 10 by each judge), with a resulting average resemblance score of 0.93/1. Note that the other behavioral episode possibly related to task re-enactment (Patient 2; sleepwalker who experienced the “cross” dream) was evaluated below 5/10 (0.6/1).

**Figure 5:**
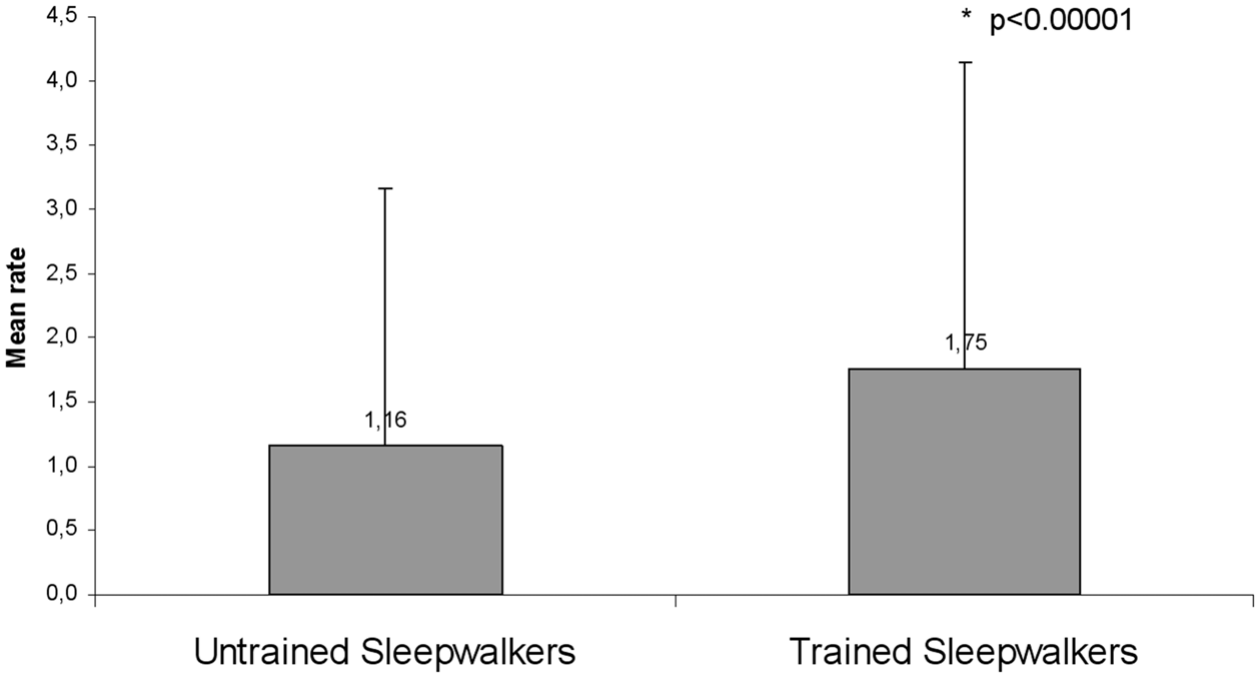
Average resemblance between the motor episodes during sleep and the trained motor sequence, when separating sleepwalkers trained on the task (one or two days before the recording) and those who had not been trained on the task. Trained sleepwalkers exhibited more movements similar to the trained task compared to motor episodes from untrained sleepwalkers (p = 0.000006). This result was still significant when removing excerpt 93 (the putative replay; p = 0.0002). Standard deviations are shown on the figure.

We also computed the average resemblance score (with the trained motor sequence) separately for sleepwalking episodes from patients who were trained on the task (47 episodes corresponding to 14 patients) and for episodes from patients not trained on the task (68 episodes corresponding to 30 patients). The average resemblance was higher in the trained compared to the untrained group (p<0.00001) (Figure 5).

A summary of the movements' description of the sleepwalking videos by the eleven judges is provided in Text S1.

## Discussion

To our knowledge, the present findings represent the first direct and unambiguous demonstration of overt behavioral replay of a recently learned skill during human sleep.

Several lines of evidence support our claim that the behavior observed during an episode of sleepwalking (Patient 1) is a true re-enactment of the trained serial reaction time task. First, there is a striking similarity between the hand movements displayed by Patient 1 and those of the waking subject performing the sequence from memory: 11 judges blind to the experimental procedure scored this episode as closely similar to the trained sequence, after careful observation of 113 video sleepwalking episodes (Figure S2). Second, the complex motor patterns performed by Patient 1 during slow-wave sleep are consistent with activation of large-scale, distributed brain networks during slow-wave sleep-associated replay, as previously suggested by animal studies [27]. Finally, our finding that the behavioral replay concerned only a part of the sequence is in line with the fragmentary replay observed in animal studies and in human dream studies [28].

Actually, the probability of observing overt behaviors in patients with REM sleep behavior disorder and in sleepwalkers is low, making our finding of overt replay highly remarkable. Indeed, patients with REM sleep behavior disorder exhibit complex, purposeful behaviors during only 0.1% to 20% of the total time spent in REM sleep [29]. In sleepwalkers, overt behavior is even rarer, corresponding to a mean of 0.7% (or ∼one minute) of non REM sleep per night. These estimations suggest that the observed behavior in the first sleepwalking patient may emerge from “a strong pressure”, probably arising from active neural processes of re-shaping and strengthening of the intensively trained information during post-training sleep, related to off-line recapitulation of the newly acquired cognitive schema. Furthermore, because elements from recent memory are frequent in dreams [30] and may correlate with learning [31], it is likely that mental replay did occur in other trained patients in the absence of overt behavioral expression. Further support for a replay of waking motor behavior during subsequent episodes of sleepwalking is also provided by the fact that sleepwalkers who performed the task were more likely to show movements that were on averaged rated as more similar to the trained motor task compared to sleepwalkers who were not trained on the task (Figure 3).

Such complex motor patterns performed by the sleepwalker showing evidence of experience-dependent behavior during slow-wave sleep is consistent with data in animal studies, showing activation of large-scale, distributed brain networks during slow-wave sleep-associated replay [27]. The evidence for a behavioral replay reflecting only a part of the sequence is in line with the fragmentary replay observed in animal studies and in human dream studies [28].

We would like to suggest that the study of motor behaviors in sleepwalkers provides highly valuable information about cognitive and motor processes occurring during sleep. Indeed, sleep macro- and micro-structure (e.g. rapid eye movement density, EEG) is normal in patients with REM sleep behavior disorder and sleepwalkers [29], [32]. Sleepwalking results from concomitant local sleep in the frontoparietal associative cortices and local arousal in motor and cingular cortices, as shown by recent functional brain imaging and deep brain monitoring in humans during sleepwalking [33], [34]. Therefore, except for their motor aspects, the mechanisms regulating sleep in sleepwalkers are mostly intact. Furthermore, like healthy controls, both sleepwalkers and the patients with REM sleep behavior disorder improved their performance, thus showing spared overnight memory consolidation processes.

Our work therefore demonstrates that parasomnias such as sleepwalking and REM sleep behavior disorder are useful neurological models for studying cognitive functions during sleep, and may for example motivate further investigations on the respective contribution of non REM and REM sleep on learning and brain plasticity.

## Supporting Information

Figure S1 **Improvement in tapping speed for all groups.** Mean reaction times (RT) showing a reduction during training session and a further decrease between pre and post-sleep testing. Persistence of increased RT for the random blocks relative to decreased RT for the structured blocks within the same session of testing (before-sleep and post-sleep sessions) confirmed sequence-specific learning with training. Faster RT in sleepwalkers may be explained by their lower mean age, as compared to REM SLEEP BEHAVIOR DISORDER patients (sleepwalkers: 34.42±15.35; REM SLEEP BEHAVIOR DISORDER: 66.45±6.46 yrs). R  =  random sequence.
(TIF)

Figure S2 **Evaluation of the resemblance between the sleepwalking episodes and the sequence performed during wakefulness.** Each point corresponds to one of the 113 video clips. Most sleepwalking episodes were rated below 2/10 by the 11 judges. Only one clip (number 93) obtained a mean rate greater than 5/10: this clip is the putative replay performed by Patient 1.
(TIF)

Video S1 Execution of the structured sequence in the training setting by a wake control (Part 1); execution of the sequence from memory by a wake control lying in a bed (Part 2); overt replay of a part of the structured sequence during slow-wave sleep in a sleepwalker (Part 3).
(WMV)

Text S1 (DOC)
