# Supplementary figures and images for: Evidence for the Re-Enactment of a Recently Learned Behavior during Sleepwalking
Source: PLoS One. 2011 Mar 21;6(3):e18056. doi: 10.1371/journal.pone.0018056 (PMC3061883; doi:10.1371/journal.pone.0018056)

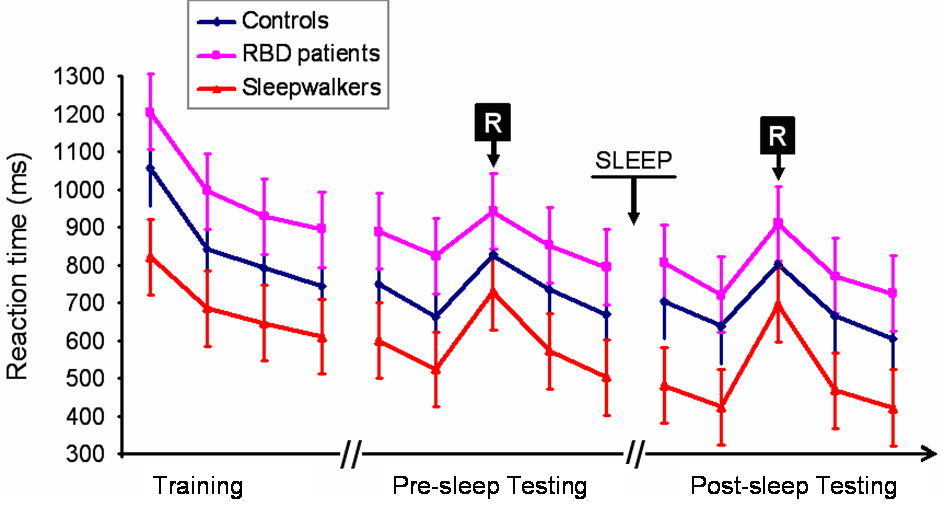

Supplement: Figure S1 — Improvement in tapping speed for all groups. Mean reaction times (RT) showing a reduction during training session and a further decrease between pre and post-sleep testing. Persistence of increased RT for the random blocks relative to decreased RT for the structured blocks within the same session of testing (before-sleep and post-sleep sessions) confirmed sequence-specific learning with training. Faster RT in sleepwalkers may be explained by their lower mean age, as compared to REM SLEEP BEHAVIOR DISORDER patients (sleepwalkers: 34.42±15.35; REM SLEEP BEHAVIOR DISORDER: 66.45±6.46 yrs). R = random sequence. (TIF) [file pone.0018056.s001.tif]

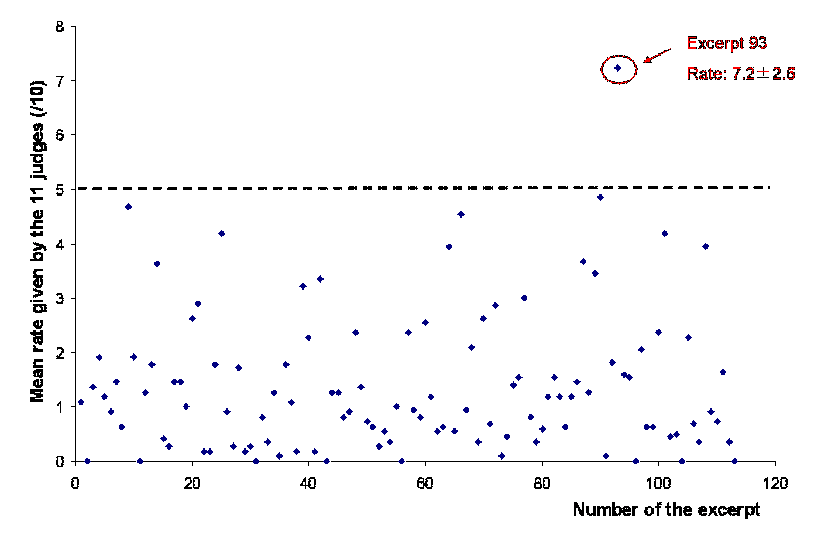

Supplement: Figure S2 — Evaluation of the resemblance between the sleepwalking episodes and the sequence performed during wakefulness. Each point corresponds to one of the 113 video clips. Most sleepwalking episodes were rated below 2/10 by the 11 judges. Only one clip (number 93) obtained a mean rate greater than 5/10: this clip is the putative replay performed by Patient 1. (TIF) [file pone.0018056.s002.tif]
