# Supplementary material for: Evidence for the Re-Enactment of a Recently Learned Behavior during Sleepwalking
Source: PLoS One. 2011 Mar 21;6(3):e18056. doi: 10.1371/journal.pone.0018056 (PMC3061883; doi:10.1371/journal.pone.0018056)
Supplement: Text S1 — (DOC) [file pone.0018056.s004.doc]

**Evidence for the re-enactment of a recently learned behavior during sleepwalking**

Delphine Oudiette*^1,3^, MSc, Irina Constantinescu* ^4,5^, MD, Laurène Leclair-Visonneau^1,3^, MD, Marie Vidailhet^2,3^, MD, Sophie Schwartz ^4,5^, PhD, and Isabelle Arnulf^1,2,3^, MD, PhD

* These authors contributed equally to this work

^1^ Sleep Disorders Unit, Pitié-Salpêtrière Hospital, APHP, Paris, France

^2^ Inserm, U975, Paris, France

^3^ Université Pierre et Marie Curie-Paris6, Centre de Recherche de l'Institut du Cerveau et de la Moelle epiniere, UMR-S975, Paris, France

^4^ Department of Neuroscience, and Geneva Neuroscience Center, University of Geneva, Switzerland

^5^ Swiss Center for Affective Sciences (NCCR), University of Geneva, Switzerland

Article submitted to: Plos One

Version: 1

Correspondence to: Dr Isabelle Arnulf

Unité des Pathologies du Sommeil , Hôpital Pitié-Salpêtrière

47-83 boulevard de l’Hôpital

75651 Paris Cedex 13

Phone: 01 42 16 77 02

**SUPPLEMENTAL INFORMATION**

Video recordings of 113 sleepwalking episodes were edited into short clips and unique numerical identifiers were inserted before each clip. The order of the episodes was randomized and the clips were then copied one after the other into a single movie file that was shown to 11 judges, blind to the experimental hypotheses and conditions. The judges were asked to answer five yes/no questions for each of the video clip, as well as final evaluation of the global resemblance with the clip of learned sequence performed from memory during wakefulness (recorded with the same infrared camera). This procedure provided an independent assessment of the similarity of several motor aspects in each sleepwalking episode and the learned motor sequence.

**Questions to judges**

**Question 1.** During this episode, did the subject perform any movements involving the superior limbs (e.g. arm, hand movements)?

If the answer to Question 1 was no, then the judge could skip the remaining questions and watch the next clip. If the answer was yes, then the judge had to answer the next 5 questions.

**Question 2.** During this episode, did the observed behavior include movements of both superior limbs (left AND right hand, arm movements)?

**Question 3.** During this episode, were the movements of superior limbs ample (i.e., more than 10 cm amplitude)?

**Question 4.** During this episode, did the movements of superior limbs include the use of the environment (e.g. touch the electrodes, grab an object on the bed table).

**Question 5.** During this episode, did the movements of superior limbs include a crossing of the body midline?

After the 5 yes/no questions, the judges were asked to evaluate the similarity of the current episode with the learned motor sequence on a scale ranging from 0 (no resemblance at all) to 10 (very high resemblance).

Based on the answers provided by the 11 judges, our database of 113 sleepwalking episodes contained: 92 clips with at least one movement of superior limbs, 60 clips with movements of both superior limbs, 60 clips with ample movements of superior limbs, 12 clips with alternating movements of the superior limbs and 5 clips with a crossing of the midline.

Only 10 clips (9%) showed, at the same time, ample and alternating movements of both right and left superior limb, confirming that a behavior resembling to the learned sequence is not typical during sleepwalking episodes.

Only one sleepwalking episode was rated with a resemblance score above 5 (7.2±2.6, or 0.93 when converting the range of responses for each judge to a 0-1 scale), thus pointing to one of the episodes as a potential replay of the learned motor sequence (Supplemental Figure 2).
